# Associations between social cognition, skills, and function and subclinical negative and positive symptoms in 22q11.2 deletion syndrome

**DOI:** 10.1186/s11689-016-9175-4

**Published:** 2016-11-16

**Authors:** A. Vangkilde, J. R. M. Jepsen, H. Schmock, C. Olesen, S. Arnarsdóttir, W. F. C. Baaré, K. J. Plessen, M. Didriksen, H. R. Siebner, T. Werge, L. Olsen

**Affiliations:** 1 Institute of Biological Psychiatry, Mental Health Centre Sct. Hans, Copenhagen University Hospital, Mental Health Services, Capital Region of Denmark, Boserupvej 2, 4000 Roskilde, Denmark; 2 The Lundbeck Foundation Initiative for Integrative Psychiatric Research, iPSYCH, Aarhus, Copenhagen Denmark; 3 Child and Adolescent Mental Health Center, Copenhagen University Hospital, Mental Health Services, Capital Region of Denmark, Bispebjerg Bakke 30, 2400 Copenhagen NV, Denmark; 4 Lundbeck Foundation Center for Clinical Intervention and Neuropsychiatric Schizophrenia Research (CINS) and Center for Neuropsychiatric Schizophrenia Research (CNSR), Copenhagen University Hospital, Mental Health Services, Capital Region of Denmark, Ndr. Ringvej 29-67, 2600 Glostrup, Denmark; 5 Department of Pediatrics, Aarhus University Hospital, Palle Juul-Jensens Blvd. 99, 8200 Aarhus N, Denmark; 6 deCODE genetics, Amgen, Sturlugata 8, 101 Reykjavik, Iceland; 7 Department of Clinical Medicine, University of Copenhagen, Blegdamsvej 3B, 2200 København N, Denmark; 8 H. Lundbeck A/S, Ottiliavej 9, 2500 Valby, Denmark; 9 Danish Research Centre for Magnetic Resonance, Centre for Functional and Diagnostic Imaging and Research, Copenhagen University Hospital, Kettegaard Allé 30, 2650 Hvidovre, Denmark; 10 Department of Neurology, Copenhagen University Hospital Bispebjerg, Bispebjerg Bakke 23, 2400 Copenhagen NV, Denmark

**Keywords:** 22q11 deletion syndrome, Schizophrenia, Social cognition, Social skills, Social function, Theory of mind, Emotional recognition task, Negative symptoms, Positive symptoms

## Abstract

**Background:**

Identification of the early signs of schizophrenia would be a major achievement for the early intervention and prevention strategies in psychiatry. Social impairments are defining features of schizophrenia. Impairments of individual layers of social competencies are frequently described in individuals with 22q11.2 deletion syndrome (22q11.2DS), who have high risk of schizophrenia. It is unclear whether and to what extent social impairments associate with subclinical negative and positive symptoms in 22q11.2DS, and which layer of social impairments are more correlated with schizophrenia-related symptoms. The aims of this study were to conduct a comprehensive investigation of social impairments at three different levels (function, skill, and cognition) and their interrelationship and to determine to what degree the social impairments correlate to subclinical levels of negative and positive symptoms, respectively, in a young cohort of 22q11.2DS not diagnosed with schizophrenia.

**Methods:**

The level of social impairment was addressed using questionnaires and objective measures of social functioning (The Adaptive Behavior Assessment System), skills (Social Responsiveness Scale), and cognition (The Awareness of Social Inference Test and CANTAB Emotional Recognition Task), and the presence of subclinical symptoms of schizophrenia were evaluated using the Structured Interview for Prodromal Syndromes in a cross-sectional case-control study of 29 cases and 29 controls, aged 12 to 25 years. Association between social impairment and negative and positive symptoms levels was examined in cases only.

**Results:**

Subjects with 22q11.2DS were highly impaired in social function, social skills, and social cognition (*p* ≤ 6.2 × 10^−9^) relative to control peers and presented with more negative (*p* = 5.8 × 10^−11^) and positive (*p* = 7.5 × 10^−4^) symptoms. In particular, social functional and skill levels were highly associated with notably subclinical negative symptoms levels.

**Conclusions:**

This study shows strong correlations between levels of social impairments and subclinical negative and positive symptoms. However, longitudinal studies are required to show if social impairments represent early disease manifestations. If parental or self-reporting suggests severe social impairment, it should advocate for clinical awareness not only to social deficits per se but also of potential subclinical psychosis symptoms.

## Background

The 22q11.2 deletion syndrome (22q11.2DS) results from a hemizygous deletion on chromosome 22q11.2 and is the most common microdeletion syndrome in humans, with an estimated prevalence of 1:4000 [1, 2]. The syndrome is associated with a variety of clinical manifestations of highly variable expressions [3] including a high prevalence of psychiatric disorders. Notably, schizophrenia spectrum disorders are seen in up to 41% of the clinically identified adult 22q11.2 deletion carriers [4–9], and large cross-sectional case-control studies in schizophrenia have reported odds ratio estimates for the 22q11.2 deletion between 15 and 44 [10–13]. Moreover, an epidemiological population study has shown that the risk of schizophrenia spectrum disorders among persons diagnosed with 22q11.2DS is approximately eight times the risk of the general population [14].

The clinical presentation of schizophrenia in 22q11.2DS has considerable overlap with clinically defined idiopathic forms of schizophrenia [7, 15] and as such, 22q11.2DS may represent a biological homogeneous “at risk group”, that can further the understanding of both the behavioral and biological mechanisms underlying development of schizophrenia in the general population. Also, since more 22q11.2 deletion carriers are being identified at a young age, they may provide insights into the early signs of schizophrenia pathology.

Social impairments are core features of schizophrenia [16] and can be categorized into three multidimensional constructs, nested within each other. The most narrow construct can be defined by social cognitive deficits, which underlie the more widely concept of social skills that again contributes to the broad construct of social function [17]. “Social cognition” refers to the mental processes involved in perceiving, attending to, remembering, thinking about, and making sense of the people in our social world [18] and includes emotional processing and theory of mind (ToM) [19]. “Social skills” refers to verbal and non-verbal behaviors with complex cognitive abilities necessary to engage in successful interpersonal interactions (e.g., initiating a conversation) [20, 21]. Lastly, “social function” is operationalized in a variety of ways, encompassing performance across everyday domains (e.g., independent living, employment, interpersonal relationships, and recreation) [22].

The reported social impairments in schizophrenia include social cognitive functions [23–29], social skills [30, 31], and social functioning [31–33]. In particular, diminished everyday functioning and social skills are associated with negative rather than positive symptoms in schizophrenia [31, 34]. Similar results have been reported for a group of children and adolescents (age 12–18 years) at clinical high risk for schizophrenia presenting with either prodromal or transient and recent-onset psychotic symptoms; a recent drop in daily functioning combined with schizotypal personality disorder or a first degree relative with a psychotic disorder [35]. Moreover, the Dutch Genetic Risk and Outcome in Psychosis (GROUP) study has shown correlations between measures of social cognitive impairments (i.e., The Degraded Facial Affect Recognition task) and negative symptoms severity in both patients with non-affective psychotic disorders and subclinical negative symptoms in their healthy siblings. This finding may suggest that there is a common origin of the social cognitive functions and negative symptoms [23].

Previous studies have reported that children and adolescents with 22q11.2DS have impairments in social functioning [36, 37], social skills [38], and social cognition, e.g., [38–41]. Diminished ToM has previously been reported to associate with the severity of psychotic symptoms in adolescences with 22q11.2DS [42] and, Chow and collaborators [43] found more impairments of ToM among 22q11.2DS adults with schizophrenia compared to 22q11.2DS adults without psychosis. However, there is still limited knowledge on the inter-relationship between different layers of social competences in 22q11.2DS and how impairments in the individual layers of the social competences relate to symptoms of schizophrenia in young 22q11.2 deletion carriers.

The aims of this study were twofold; to characterize impairments across social functioning, skills, and cognition and their inter-relationship in 22q11.2 deletion carriers who are at risk for developing schizophrenia and to investigate the strengths of correlation between these different layers of social impairments and subclinical levels of negative and positive symptoms in a young 22q11.2DS cohort without schizophrenia. We expected that the 22q11.2DS group would show deficits at all three layers of social competence that the different measures of social competences were inter-correlated and that the level of social impairments associated with the subclinical levels of negative and positive symptoms.

## Methods

### Study design

The study applies two different study designs. First, to document the level of overall social impairments, their inter-relationship, and examine the amount of psychopathology within the 22q11.2DS group, we used a cross–sectional case–control design in which each 22q11.2DS person was individually matched on sex and age with a non-related control individual. Second, we applied a case-only design to test for associations between the degree of social impairments and the levels of psychopathology.

### Participants

The total sample consisted of 29 clinically identified subjects with 22q11.2DS in whom the deletion was verified by SNP array analysis (Human OmniExpress BeadChip Illumina) and 29 control individuals. Based on the clinical diagnoses reported in the comprehensive Danish health registries, we have previously shown that the recruited cases represent the entire cohort of people diagnosed with 22q11.2DS in Denmark [44]. Current diagnoses of affective disorder, anxiety, and disturbance of activity and attention/attention–deficit disorder without hyperactivity were provided by the use of the Mini International Neuropsychiatric Interview [45] or the Mini International Neuropsychiatric Interview for Children and Adolescents [46]. The current diagnosis of schizophrenia was obtained from the Structured Interview for Prodromal Syndromes [47], and IQ below 70 determined from Reynolds Intellectual Screening Test (RIST) [48] was used as an indication of current intellectual disability. We used the clinical threshold of 15 derived from the Social Communication Questionnaire lifetime form [49, 50] to indicate the presence of current autism spectrum disorders. Lifetime diagnoses for psychiatric disorders were obtained from the Danish Psychiatric Central Register that holds information on all Danish psychiatric inpatient facilities since April 1, 1969, and for outpatient facilities since 1995 [51]. The diagnoses in Denmark are assigned according to the International Classification of Diseases (ICD) system, and as outlined by several investigators, the validity of the register diagnoses is good [52–54]. In Denmark, every citizen has a unique civil registration number, which is given at birth or upon immigration [52]. This civil registration number can be linked to information recorded in the Danish Psychiatric Central Register, which automatically obtains records on all clinical discharge diagnoses given in Denmark [55]. Since health care is free of charge, all Danish citizens can get treatment for all their illnesses. This ensures that the psychiatric registry reflects the disorders that Danish citizens are treated for during their lifetime. The sample characteristics are shown in Table 1.

**Table 1:** Demography, lifetime and current history of psychiatric disorders

	22q11.2DS (*n* = 29)		Controls (*n* = 29)	
Mean age (SD)	15.7 (2.8)		16.0 (2.8)	
Male/female ratio	19:10		19:10	
Caucasian ancestry	29		29	
3 megabase deletion	29		0	
Intelligence quotient (range)^a^	79.52 (35–113)		108.76 (87–129)	
Psychiatric diagnoses %	Lifetime^b^	Current^a–e^	Lifetime^b^	Current^a–e^
Schizophrenia spectrum disorders (F2x)^c^	0	0	0	0
Affective disorders (F30-F33)^d^	3.4	3.4	0	0
Anxiety and phobia (F4x)^d^	3.4	34.5	0	13.8
Intellectual disability (F7x)^a^	17.2	20.1	0	0
Autism spectrum disorders (F84.x)^e^	13.8	13.8	0	0
Disturbance of activity and attention/attention-deficit disorder without hyperactivity (F90.0/98.8C)^d^	3.4	3.4	0	0

^a^Intelligence quotient score was derived from the Reynolds Intellectual Screening Test (RIST) [48]. Indication of current intellectual disability was indexed by RIST IQ below 70

^b^Lifetime diagnoses according to International Classification of Diseases (ICD-10) obtained from the Danish Psychiatric Central Register [51]

^c^Current diagnosis of schizophrenia was obtained from the Structured Interview for Prodromal Syndromes

^d^Current diagnoses of affective disorder, anxiety, and disturbance of activity and attention/attention-deficit disorder without hyperactivity were provided by use of the Mini International Neuropsychiatric Interview [45, 82] or the Mini International Neuropsychiatric Interview for Children and Adolescents [46]

^e^Indications of current autism spectrum disorders (ASD) was determined from parental reporting using the Social Communication Questionnaire lifetime form. Clinical cut-off of 15 indicates ASD [49, 50]

### Recruitment

All participants were enrolled in the Danish research initiative of 22q11.2DS [44]. The individuals with 22q11.2DS were recruited from the Pediatric Department, Aarhus University Hospital, Clinical Genetic Department of Rigshospitalet, Copenhagen, or the Danish National 22q11.2DS Association. The two clinical departments are non-specialty clinics, which monitor healthcare and/or social community issues. When clinical healthcare is needed, patients are referred to specialized clinics for treatment. Cases that had a prior or current clinical diagnosis of schizophrenia according to ICD-10 diagnostic criteria were not enrolled. Unrelated control participants were recruited either by letter through the National Danish Civil Registry, via public postings on www.forsøgsperson.dk, or among children of participants in the Danish Blood Donor Study. Control individuals fulfilling the criteria for psychiatric disorders including schizophrenia spectrum (ICD10 DF20-29); bipolar affective (ICD10 DF30-31); depression (ICD DF32-33); and autism spectrum disorders (ICD10 DF84); and substance abuse (ICD10 DF10); according to ICD-10 criteria were excluded based on life time history of psychiatric disorders obtained from the Danish Health Registries and current state presentation of symptoms evaluated as described above.

### Measures

#### Schizophrenia symptoms

The Structured Interview for Prodromal Syndromes (SIPS) [47] was part of a larger examination battery for psychopathology [44] and used to rate the severity of 19 schizophrenia-related symptoms. SIPS ratings are given on a 0–6 point scale within four domains: positive, negative, disorganized, and general symptoms. A rating of zero represents absence of symptoms, and six refers to extremely severe level of symptoms. Ratings of 3–5 are within the attenuated range [56]. In the current study, we focused on the positive and negative symptoms scores.

The positive symptoms score is a composite measure obtained by ratings of five items: delusional ideas, persecutory ideas, grandiosity, hallucinations, and disorganized communication. The negative symptoms score is a composite measure including six items: anhedonia or withdrawal, avolition, decreased expression of emotions, decreased experience of emotion or self, impoverished thinking, and deterioration of role functioning. The disorganized composite score includes odd behavior and appearance, bizarre thinking, trouble with focus and attention, and personal hygiene (total raw composite score range 0–24). The generalized composite score defines odd behavior and appearance, bizarre thinking, trouble with focus and attention, and personal hygiene (total raw composite score range 0–25). We used the raw sum of positive (score range 0–30) and negative (score range 0–36) symptoms scores as separate dimensional measures of psychotic symptoms. The SIPS interview was conducted by two experienced and SIPS certified clinicians (AV and HS) who demonstrated good inter-rater reliability (*k* = 0.73).

#### Intellectual functioning

Reynolds Intellectual Screening Test (RIST) [48] was used to assess the intelligence level. The RIST consists of (1) a verbal “guess what” subtest where the guess is based on subject cues and (2) a non-verbal “odd item out” subtest (i.e., which figure does not fit in according to classifications or spatial relations). Questions/figures are given from floor level in order of increasing difficulty until three consecutive failures are obtained. The intelligence quotient (IQ) was based on the sum of the two subtest T scores derived from Danish test norms provided by the publisher.

#### Social functioning

The Adaptive Behavior Assessment System—Second Edition (ABAS-II) [57], which assesses adaptive functioning, was used as to assess the general everyday functioning whereas the social subdomain was used as an index of social functioning. Adaptive functioning entails the daily activities required for personal and social sufficiency defined by the expectations or standards of other people [58]. Parents or daily caretakers were respondents for participants under 18 years of age. The questionnaire was self-administered for participants over 18 years (with aid from a caretaker if necessary). A general adaptive composite score (ABAS_composite_), which includes three subdomains: conceptual, social, and practical was derived from raw scores of the nine sub-scales in ABAS-II and the social subdomain raw score (ABAS_social_) specifically address the functioning in social and leisure situations. Danish norms do not exist, but ample reliability and validity data are included in the Danish version of the ABAS-II manual ([59], page 372).

#### Social skills

Parent ratings of the Social Responsiveness Scale (SRS) [60] were used to assess social skills. SRS is a 65-item questionnaire on social behavior the past 6 months, which focuses on the ability to perceive and process social information and respond appropriately in interpersonal interactions [60]. SRS applies a 4-point Likert scale (0 = not true, 1 = sometimes true, 2 = often true, 3 = almost always true) to rate how often the subject engages in specific social and communicative behaviors. SRS provides an overall raw-score (SRS_total_). This scale has previously shown discriminant validity at the group level in Danish case–control studies of autism [61, 62].

#### Social cognition—theory of mind (ToM)

The Danish translation of the Awareness of Social Inference Test—Part A2 Social Inference minimal (TASIT) [63–65] was used to test for ToM abilities, which has shown discriminant validity at the group level in a Danish schizophrenia case–control study [63]. TASIT is composed of 15 video clips showing professional actors performing everyday interactions using sincere, simple, and paradoxical sarcasm. Sincere clips show congruence between what is literally said and the accompanying paralinguistic and facial cues. Simple sarcastic clips show incongruence between the spoken word and paralinguistic and facial cues and can only be understood if cues are correctly interpreted and contradictions are recognized. Correct interpretation of the paradoxical sarcastic clips requires the ability to understand paralinguistic cues facial expressions, tone of voice, gesture, etc. [64]. Each film clip is followed by four questions addressing different aspects of the communicative intentions of the persons (what they were doing, saying, thinking, and feeling). The maximum total TASIT score (TASIT_total_) is 60 (ranging from 0–20 for each type of interaction; sincere, simple, and paradoxical sarcasm).

#### Social cognition—emotion recognition

The Emotion Recognition Task (ERT) from the Cambridge Neuropsychological Test Automated Battery [66] was used to test subject’s ability to recognize emotions in facial expressions. The ERT consists of computer-morphed images of facial features from a real individual showing specific emotions. Each participant was shown 90 images in total, displayed for 200 ms, and followed by an immediate mask stimulus. The participant then chose which emotion (i.e., sadness, fear, disgust, happiness, surprise, and anger) best described the displayed emotion. The outcome measures are percentage correctly identified emotions for the each of the six individual emotions, together with a total percent score (ERT_total_).

### Statistical analyses

All statistical analyses were performed using R version 3.2.2 [67]. Normal distribution and homogeneity of variances were evaluated from histograms, Shapiro–Wilk normality test and Levene’s test, respectively. Additional assumptions for ANCOVA and regression analysis were examined as follows: (a) linearity of the relationship between the functional measures and covariate or symptom level was seen from examination of scatter plots, (b) independence and normality of error terms was supported by inspections of histograms and P–P plot of the residuals, (c) homoscedasticity was inferred from residuals plots, (d) homogeneity of regression slopes were examined by evaluation of the pairwise interaction terms group*sex, age*sex, and age*group and accepted in the absence of an significant interaction, and (e) absence of multicollinearity was determined by (i) pairwise correlations with *r* < 0.8, (ii) tolerance measures *T* > 0.2, and (iii) variance inflation factors (VIF) <10.

Two-tailed independent sample *t* test and chi-squared test were used to compare the basic demographic factors (age, sex, and IQ) and between groups.

#### Case–control comparisons

The total raw scores of ABAS_composite_, ABAS_social_, SRS_total_, TASIT_total_, and ERT_total_ were transformed into *z*-scores, using the mean and standard deviation from the control group. A negative *z*-score for ABAS_composite_, ABAS_social_,TASIT_total_, and ERT_total_ and a positive *z*-score for SRS_total_ indicates that 22q11.2DS cases performed worse than the average control individual. Missing values reduced sample size for the 22q11.2DS and control groups across domains; ABAS_composite_ and ABAS_social_(*n* = 27 and 28); SRS_total_ (*n* = 25 and 28); TASIT_total_ (*n* = 28 and 29); ERT_total_ (*n* = 28 and 29).

To comply with the requirement for normal distribution, SIPS scores for all four subdomains were square root transformed. However, only SIPS data for negative and positive were normally distributed and only in the 22q11.2DS group. Therefore, we used Wilcoxon rank-sum test to test for case–control differences in SIPS scores. Sensitivity analyses were performed post hoc to address if 22q11.2DS participants with attenuated symptoms (*n* = 6) were driving group differences.

We used two-way ANCOVA to test the main effect of ABAS_composite_, ABAS_social_, and ERT_total_ using their *z*-score as dependent variable, participant (22q11.2 deletion carrier or control subject), sex as factor, and age as covariate. SRS_total_ and TASIT_total_ did not meet the assumptions of normal distribution and homogeneity of the variances. Therefore, we applied Wilcoxon rank-sum test to test for group differences (22q11.2DS versus control subjects) and sex differences. A *p* value of 0.007 corresponds to Bonferroni adjustment for multiple testing of the seven group comparisons. Age effects of SRS_total_ and TASIT_total_ were addressed using Spearman’s correlation analysis. Wilcoxon rank-sum test was applied for post hoc analyses of group differences in subdomains for TASIT and ERT and to test for differences in the ratings and test scores between the 22q11.2DS individuals with and without IQ <70 as an index of intellectual disability.

Pearson’s product-moment correlation or Spearman’s rank correlation rho analyses were used to test for significant inter-correlations between the four measures of social impairments (ABAS_social_, SRS_total_, TASIT_total_, and ERT_total_) and to explore the possible effect of IQ and ABAS_composite_ on the individual performance in the four separate measures. Spearman’s rank correlation was used when one of the pairwise measures did not meet the assumptions of normality and/or homoscedasticity (i.e., SRS_total_ and TASIT_total_). These analyses were conducted separately for the case and the control groups.

#### Case-only analyses

The relationship between social competences and negative and positive symptom levels was independently accessed within the 22q11.2DS cohort only using linear regression analyses. Disorganized and generalized symptoms were sparse and therefore not assessed. Square root transformed total negative and positive symptoms were treated as dependent variables and centralized *z*-scores derived for measures of ABAS_social_, SRS_total_, TASIT_total_, or ERT_total_ were included as predictor variables. The centralized *z*-scores were based on the mean and standard derivation within the 22q11.2DS group. The basic model included age, sex, and IQ and served as offset for four separate extended models for negative symptoms that a part from age, sex, and IQ also included centralized *z*-scores for ABAS_social_, SRS_total_, TASIT_total_, or ERT_total_, respectively. Two extended models that included both ABAS_social_ and SRS_total_ and ABAS_social_ and TASIT_total_, respectively, were also investigated. Only three separate models for positive symptoms, which included age, sex, IQ and either centralized *z*-scores for ABAS_social_, SRS_total_, and TASIT_total_ were evaluated, as ERT_total_ did not meet the assumption for linearity with positive SIPS scores. If the individual model was overall significant, we used analysis of variance to examine if this model explained significantly more of the variation in SIPS scores compared to a reduced model that include age, sex, and IQ or age, sex, IQ, and ABAS_social_ in the case of the extended models. Bonferroni-corrected significance threshold was set to *p* = 0.006 (evaluation of eight linear models).

## Results

### Demography

There was an overall nominally significant sex difference in mean age (*t*(56) = 2.2, *p* = 0.03). The 22q11.2DS group had mean IQ well below the general population mean and differed significantly from the mean IQ in the control subjects (*t*(56) = 7.4, *p* = 2.14 × 10^−10^). A total of six individuals with 22q11.2DS had IQ below 70 but within the mild (IQ 50–69) to moderate range (IQ 35–49). The 22q11.2DS cohort presented with reduced everyday functioning as indicated by a mean ABAS_composite_ rating, which were more than two standard derivations below the mean of the control group. There was a significant group *F*(1,51) = 62.3, *p* = 2.14 × 10^−10^, age *F*(1, 51) = 20.9, *p* = 3.16 × 10^−5^, and sex *F*(1, 51) = 9.8, *p* = 0.003 effect for ABAS_composite_ ratings. Table 1 shows that there was consistency between the lifetime and current prevalence of all psychiatric disorders but anxiety and phobias. This is due to the presence of specific phobias (e.g. arachnophobia) in both cases and control individuals, which people rarely seek health care for.

### Schizophrenia-related symptomatology

Overall, the 22q11.2DS cohort had significantly elevated mean SIPS scores for negative, positive, and disorganized but not generalized symptoms, relative to control peers (Table 2). The majority of the individuals with 22q11.2DS (*n* = 27) had negative symptoms scores of two standard derivations above the mean of the control group, and six of these individuals presented at least one attenuated psychotic symptom (i.e., SIPS score above three in one or more positive symptoms). Only three individuals with 22q11.2DS presented with elevated disorganized symptoms. A sensitivity analysis leaving out the six individuals with attenuated positive symptoms provided similar nominal significant between-group difference for negative (*W* = 657, *p* = 6.51 × 10^−10^, positive (*W* = 459, *p* = 0.01), disorganized (*W* = 566, *p* = 6.35 × 10^−7^) as well as generalized (*W* = 355, *p* = 0.60) symptom scores.

**Table 2:** Characteristics of negative and positive SIPS scores and social competences in 22q11.2DS and control peers

	22q11.2DS		Control		Main effect of group		
Test	Median or mean (SD)	Min-max	Median or Mean (SD)	Min-max	*z* ^a^	*W* or *F*(*df*,*df*)	*p*
Psychopathology							
Positive symptoms^b^	3	0–12	0	0–9		625	7.5 × 10^−4^
Negative symptoms^b^	6	1–17	0.38	0–2		831	5.8 × 10^−11^
Disorganized symptoms^b^	1	0–9	0	0–2		723.5	1.2 × 10^−07^
General symptoms^b^	0	0–7	0	0–4		469.5	0.32
Social functioning							
ABAS_social_^c^	99.89 (18.6)	256–606	126.1 (9.26)	110–138	−2.77	61.02 (1,50)	2.9 × 10^−10^
Social skills							
SRS_total_^b^	64	15–122	15.17	3–44	5.00	678	6.2 × 10^−9^
Social cognition							
TASIT_total_^b^	38	16–56	52.83	37–58	−3.83	44.5	8.0 × 10^−9^
ERT_total_^c^	40.83 (12.09)	16.67–64.44	64.52 (7.75)	47.67–77.78	−2.96	88.07 (1,53)	7.39 × 10^−13^

Measures of psychopathology were obtained from the structured interview for prodromal syndromes (SIPS), The raw score obtained from the social domain of the Adaptive Behavior Assessment System (ABAS-II) was used as a measure of social function, social skills were the total score obtained from the Social Responsiveness Scale (SRS), social cognitive abilities were evaluated using total correct answers from The Awareness of Social Inference Test (TASIT) and the CANTAB Emotional Recognition Task (ERT)

^a^Mean *z*-scores for 22q11.2 group as calculated from mean control = 0 and SD = 1

^b^Group median reported; group differences determined from Wilcoxon rank-sum test

^c^Group mean reported, main effect of group accessed from two-way ANCOVA [between-group factor (22q11.2DS, controls); between subject’s factor: sex; covariate: age]

### Social competence, age, and sex

The 22q11.2DS cohort was significantly impaired on all measures of social function, skills, and cognition, relative to the control group (Table 2).

The 22q11.2DS group had mean ratings and scores of more than two standard derivations below the mean of the control individuals for both ABAS_social_ and ERT_total_. There was a statistically significant main effect of group as determined by two-way ANCOVA for ABAS_social_ and ERT_total_ (Table 2) as well as a significant effect of age, *F*(1, 51) = 10.8, *p* = 0.002, and sex *F*(1, 51) = 14.90, *p* = 0.003 for ABAS_social_ ratings. There was also a significant main effect of age, *F*(1, 53) = 10.58, *p* = 0.002 but not sex *F*(1,53) = 3.26, *p* = 0.08 in ERT_total_ scores. There was no pairwise interaction between age, sex, and group for either ABAS_social_ or ERT_total_ (*p* > 0.05). Post hoc analyses showed that relative to the control group, the 22q11.2DS group was significantly less accurate in detecting the ERT subdomains of feelings of sadness, fear, disgust, anger, and happiness but not surprise (Table 3).

**Table 3:** Impairments in subdomains of social cognitive functions in 22q11.2DS relative to controls

	*W* ^a^	*p* value
ERT subdomains		
Disgust	44	6.77 x10^−9^
Sadness	90.1	4.44 × 10^−7^
Anger	96	5.95 × 10^−7^
Happiness	189	5.0 × 10^−4^
Fear	197	8.0 × 10^−4^
Surprise	340	0.3
TASIT subscales		
Paradoxical sarcasm	53	5.55 × 10^−8^
Simple sarcasm	84.5	1.16 × 10^−6^
Sincere	167.5	6.0 × 10^−4^

^a^Group differences determined from Wilcoxon rank-sum test

Wilcoxon rank-sum tests showed statistical significant differences in SRS_total_ and TASIT_total_ scores between the 22q11.2DS and control group (Table 2).

Post hoc analysis revealed that all three types of social interactions accessed using TASIT (i.e., sincere, simple sarcasm, and paradoxical sarcasm) were significantly impaired in the 22q11.2DS group compared to the control group (Table 3).

Spearman’s correlation analysis showed an age-related trend in SRS_total_ ratings (*r*_*s*_ = −0.38, *p* = 0.053) and TASIT_total_ scores (*r*_*s*_ = 0.35, *p* = 0.07) in the 22q11.2DS but not in the control group (SRS_total_: *r*_*s*_ = −0.14, *p* = 0.48; TASIT_total_: *r*_*s*_ = 0.21, *p* = 0.29). Using Wilcoxon rank-sum test, we found no sex differences in either SRS_total_ ratings among 22q11.2DS (*W* = 357, *p* = 0.83) or control individuals (*W* = 75, *p* = 0.63). Likewise, there were no sex differences in TASIT_total_ scores within the 22q11.2DS cohort (*W* = 73, *p* = 0.43) or the control group (*W* = 85.5, *p* = 0. 68).

### Inter-relationship between the three layers of social competencies

Spearman’s correlation analysis showed significant correlations between the ABAS_social_ and SRS_total_ ratings in both the 22q11.2DS (*r*_*s*_ = −0.78, *p* = 3.174 × 10^−6^) and the control group (*r*_*s*_ = −0.53, *p* = 0.003). Nominally, significant pairwise correlations were also seen between ERT_total_ and the TASIT_total_ score within the 22q11.2 deletion carriers (*r*_*s*_ = 0.37, *p* = 0.053) but not within the control individuals (*r*_*s*_ = 0.00, *p* = 0.99). There were no significant correlations between ABAS_social_ or SRS_total_ and TASIT_total_ or ERT_total_ (*r*_*s*_ < 0.3, *p* > 0.05).

### Explorative analyses of social competencies, everyday functioning, and IQ

We used explorative pairwise correlation analyses to explore if the social impairments across the different layers were related to overall everyday functioning (ABAS_composite_) and IQ. Spearman’s correlation analysis showed significant correlations between the ABAS_composite_ and SRS_total_ ratings in both the 22q11.2DS (*r*_*s*_ = −0.79, *p* = 1.37 × 10^−6^) and the control group (*r*_*s*_ = −0.48, *p* = 0.008). Pearson’s correlation analysis showed a strong inter-relationship between ABAS_composite_ and ABAS_social_ in both groups (22q11.2DS: *r* = 0.84, *p* value = 4.48 × 10^−8^; controls: *r* = 0.86, *p* = 1.78 × 10^−9^). There was a trend in the pairwise correlations between ABAS_composite_ ratings and the ERT_total_ score among the control individuals (*r* = 0.35, *p* = 0.07) but not the 22q11.2 deletion carriers (*r* = 0.19, *p* = 0.34).

Explorative correlation analysis showed that ERT_total_ (*r* = 0.43, *p* = 0.02) but not TASIT_total_ (*r*_*s*_ = 0.16, *p* = 0.41), SRS_total_ (*r*_*s*_ = −0.02, *p* = 0.92), or ABAS_social_ (*r* = −0.04, *p* = 0.86) correlated significantly with IQ in the 22q11.2DS group. Within the 22q11.2DS cohort, there were no significant differences in mean test score of the four independent measures of social competence when we stratified the group into those with IQ below and above 70, respectively (*p* > 0.05). Similar results were obtained for the control group in which IQ did not correlate with either SRS_total_ (*r*_*s*_ = −0.15, *p* = 0.42), or ABAS_social_ (*r* = 0.02, *p* = 0.91); but there was a trend in the correlations between IQ and ERT_total_ (*r* = 0.35, *p* = 0.06). In contrast to the findings in the 22q11.2DS group, there was a nominal significant correlation between IQ and TASIT_total_ among the control individuals (*r*_*s*_ = 0.41, *p* = 0.03).

### Associations between social competence and negative and positive symptoms in 22q11.2DS case-only analyses

Linear regression analyses revealed an overall significant effect of the age, sex, and IQ corrected models on negative SIPS symptoms, which individually included ABAS_social_, SRS_total_, and TASIT_total_ and a nominally significant effect of the model including the ERT_total_ score (Table 4) within the 22q11.2DS cohort. The analysis of variance showed that the full models that included ABAS_social_, SRS_total_, and TASIT_total_ explained a significant fraction of the variation in negative symptoms than the basics models alone (Table 4) whereas the model that included ERT_total_ did not. Table 4 shows that there was an independent significant effect of ABAS_social_, SRS_total_ ratings, and TASIT_total_ but not age and sex on the 22q11.2DS cohort.

**Table 4:** Linear models for negative and positive symptoms: the effect of social function, skills, and cognition adjusted for age, sex, and IQ

		Negative symptoms				Positive symptoms		
	Model	ABAS	SRS	TASIT	ERT	ABAS	SRS	TASIT
Intercept	Estimate	3.90	3.55	3.66	4.18	2.17	1.81	1.95
	T	4.90	4.34	4.22	4.17	1.73	1.37	1.45
	Pr(>|t|)	6.76 × 10^−5^	0.0003	0.0003	0.0004	0.1	0.18	0.16
Age	Estimate	0.04	0.06	0.03	−0.01	0.17	0.19	0.11
	T	0.88	1.25	0.71	−0.20	2.20	2.23	1.43
	Pr(>|t|)	0.39	0.22	0.48	0.84	0.04	0.04	0.17
Male sex	Estimate	−0.35	−0.05	0.02	0.002	−1.18	−0.82	−0.54
	T	−1.21	−0.21	0.08	0.006	−2.61	−1.98	−1.37
	Pr(>|t|)	0.24	0.84	0.94	0.99	0.02	0.06	0.18
IQ	Estimate	−0.02	−0.03	−0.02	−0.02	−0.03	−0.03	−0.02
	T	−3.09	−3.33	−2.94	−2.16	−2.63	−2.70	−2.03
	Pr(>|t|)	0.005	0.003	0.007	0.04	0.02	0.01	0.05
Social measure	Estimate	−0.51	0.52	−0.38	−0.19	−0.64	0.63	−0,25
	t	−3.67	3.78	−2.86	−1.23	−2.93	2.89	−1.18
	Pr(>|t|)	0.001	0.001	0.009	0.23	0.008	0.009	0.25
Full model^a^	R^b^	0.42	0.44	0.39	0.22	0.29	0.28	0.15
	*F* (*df*)	5.71 (4,22)	5.93 (4,21)	5.28 (4,23)	2.92 (4,23)	3.60 (4,22)	3.47 (4,21)	2.17 (4,23)
	P	0.003	0.001	0.004	0.04	0.02	0.03	0.10
Basic^a^ vs. full model^b^	F	13.44	14.31	8.18	1.51	8.59	8.38	1.38
	p	0.001	0.001	0.009	0.23	0.008	0.009	0.25

^a^Full model: SIPS score = age + sex + IQ + social measure (i.e., ABAS_social_, SRS_total,_ TASIT_total_, or ERT_total_)

^b^Basic model: SIPS score = age + sex+ IQ

Moreover, there was a significant effect of IQ in the models that included ABAS_social_, SRS_total_, and TASIT_total_.

An extended model that included both ABAS_social_ and TASIT_total_ (*F* = 5.82; *p* = 0.03), but not a model that included both ABAS_social_ and SRS_total_ (*F* = 2.31; *p* = 0.14), explained nominally 10% more (*r* ^2^ = 0.52) of the variation in negative SIPS symptoms than the model including ABAS_social_ alone (*r* ^2^ = 0.42).

Separate linear models including ABAS_social_ or SRS_total_ explained a nominal significant part of the variance in the positive symptoms seen among the 22q11.2 deletion carriers (Table 4). In these two models, ABAS_social_ and SRS_total_ ratings contributed significantly to the model, and there was a nominal effect of IQ, age and sex (Table 4). However, the results did not survive correction for multiple testing.

## Discussion

To the best of our knowledge, this is the first study that examines multiple layers of social impairments in the same cohort of young 22q11.2 deletion carriers, the pairwise interrelationship between the different layers of social impairments, and their relationship with schizophrenia-related symptoms. We found impairments across all layers of social competences in this young non-psychotic 22q11.2DS cohort, and as anticipated, we saw that the level of impairment in each social layer was associated with the severity of predominately subclinical negative symptoms.

### Social impairments within and across different layers

In line with previous studies of 22q11.2DS, we found strong deficits in social function [36, 37﻿, 68] and social skills [69, 70]. With a mean total SRS score of 64, we saw the same magnitude of impairment’s for comparable age groups as that previously reported by Ho and collaborators (mean SRS total score of 66–72). In contrast to our expectations, the inter-relationship among the measures varied and only the measures of social function and social skills were highly correlated. Hence, our results suggest that deficits in other cognitive functions than emotional recognition and ToM are also important for the more global social skills and functional abilities. The lack of strong correlations between the four social test variables and everyday functioning and IQ, respectively, indicated that overall, the social impairments across the different layers of interest were not solely accounted for by the overall reduced everyday (ABAS_composite_) or intellectual (IQ) functioning within our 22q11.2DS cohort.

### Social impairments and negative and positive symptoms

Our data showed that 22q11.2 deletion carriers who had worse social functioning (i.e., those who had lower ratings on ABAS_social_) and who had worse social skills (i.e., higher SRS_total_ ratings) had significantly more subclinical negative and positive symptoms. Notably, the ratings of social functioning and social skills emerged as those most associated to both negative and positive symptoms, which is consistent with findings in clinical high-risk subjects [31]. Social functioning has previously been shown to deteriorate from childhood into early adolescence among 22q11.2 deletion carriers who later developed schizophrenia [71]. In line with our results, others have also found that worse scores on parent-reported sociability, peer relations, and interests were significantly associated with higher levels of schizotypy symptoms in 22q11.2DS [72]. A previous study has also reported that adolescents with 22q11.2DS and psychotic symptoms have more social withdrawal and less adaptive socialization skills in comparison with young 22q11.2DS individuals without psychotic symptoms [73]. Taken together, these lines of evidence indicate that a drop in social functioning and social skills may predate the onset of psychosis and schizophrenia in 22q11.2DS.

ToM deficits have previously been described for a cohort of 22q11.2DS adults with schizophrenia in comparison with a 22q11.2DS cohort without schizophrenia [43]. ToM impairments (mean TASIT *z*-score of approximately −1.75 compared to control peers) have also been reported in a mixed cohort of 22q11.2DS adolescents where some presented with psychotic illness [42]. Jalbrzikowski and collaborators showed that TASIT scores were correlated with both positive and negative SIPS symptoms levels. Similarly, we found pronounced deficits in ToM as well as a strong association between TASIT_total_ scores and level of subclinical negative but not in positive symptoms in our cohort of young 22q11.2DS individuals. We also saw that ToM deficits were apparent in all three TASIT interaction types, with the more difficult interaction type (i.e., paradoxical sarcasm) showing the most significant difference between 22q11.2 deletion carriers and control peers. Due to the low levels of positive symptoms in the cohort, our data did not meet the assumption of linearity between TASIT scores and positive symptom levels. The discrepancies between our data and the results previous reported by Jalbrzikowski et al. (2012) on positive symptoms may result from difference in schizophrenia prevalence between the two 22q11.2DS cohorts under investigation. In particular, the average levels of positive symptoms differ by threefold between the two 22q11.2DS studies.

In line with previous reports [38–41], we found that the 22q11.2DS group had strong emotional recognition impairments with difficulties in perceiving emotions from facial expressions across the panel of negative emotions (i.e., anger, fear, and disgust), but that the ability to identify expressions of surprise was conserved. In contrast to these previous reports, our 22q11.2DS cohort showed reduced ability to recognize happy facial expressions. However, the CANTAB ERT test applies morphed images with fast cover up times to avoid ceiling effects. As a consequence, the CANTAB ERT test may be more sensitive to more subtle deficits compared to other emotion recognition tasks, and the inter-study discrepancies may relate to differences in the tools applied between studies.

A recent meta-analysis has shown moderate associations between impairments in emotional recognition, and the severity of negative symptoms in idiopathic schizophrenia [74] and findings from the Dutch Genetic Risk and Outcome in Psychosis (GROUP) study suggest that social cognitive deficits and negative symptoms are etiologically related [23]. Although we only observed a minor association between emotional recognition deficit and the severity of subclinical negative symptoms, in our non-psychotic 22q11.2DS group, we did observe a stronger association between the negative symptoms and the other social cognitive function accessed by TASIT. This could indicate that there is a shared origin of these phenomenon’s in 22q11.2DS. Moreover, we cannot exclude the possibility that a pathological progression of negative symptoms may result in stronger correlations between these phenomena if some of the 22q11.2 deletion carriers develop schizophrenia. This would provide stronger confirmation on the etiologically relatedness between the social cognitive deficits and the negative symptoms. Taken together, these lines of evidence suggest that 22q11.2DS may be a valuable genetic model for schizophrenia per se or for the subgroup of schizophrenia patients more highly affected by negative symptoms.

Similar to findings in idiopathic schizophrenia, e.g., [74], the social impairments in our 22q11.2DS cohort were not accounting for all of the variation in symptom severity. Hence, our results suggest that deficits in other cognitive functions are contributing to the mechanisms driving the negative and particular positive symptomatology. Moreover, the extended models that included ABAS_social_ and SRS_total_ did not improve the predictability of the linear models compared to the model for ABAS_social_ alone. This suggests that the effect of social skills on the negative symptom levels were captured by the measure of social function (ABAS_social_). In contrast, when the combined effect of both ABAS_social_ and TASIT_total_ were considered the model accounted for a significant greater proportion of the variation in negative symptoms compared to the model for ABAS_social_ alone. Together with the lack of strong inter-relationship between the ABAS_social_ rating and the TASIT_total_ score, this may suggest that the effect of ToM on the negative symptom levels is mediated by slightly different mechanisms that are not fully encompassed within the measure of social function (ABAS_social_).

### Clinical implications

Identification of the early signs of schizophrenia is mandatory for early intervention and prevention strategies in psychiatry. Emerging evidence from studies in idiopathic schizophrenia suggests that psychosocial interventions are effective in improving functionality [21, 75–77] and that early intervention may improve treatment outcome and even reduces the disease transition rate [78].

From our study, the association between social impairments and subclinical negative and positive symptom severity emphasizes the importance of clinical awareness of potential subclinical psychosis symptoms when caregiver or self-reporting suggests substantial social impairments. Based on these findings, we speculate if psychosocial interventions could be efficient in improving functioning and perhaps even reduce the severity of schizophrenia symptoms in 22q11.2DS.

Intervention models for targeted psychosocial training in 22q11.2DS have already been proposed [79, 80] and cognitive enhancement therapy of tailored exercises, which include perspective taking and identification of non-verbal social clues, has been shown to improve social cognitive abilities in adolescents with 22q11.2DS [80]. Also, the training scheme applied by Glaser et al [79] which targets focusing on the eyes, emotion recognition and understanding, and working memory has shown short-term improvements in these functions. However, the long-term effect on psychopathology of these interventions is still to be evaluated. Due to the lack of strong correlations between the social cognitive abilities and the more applied social skills and functioning, seen in our study, we speculate if psychosocial training should target both the cognitive domain and the applied skills in order to improve the overall functioning or mitigate symptom severity in 22q11.2DS individuals. Hence, longitudinal studies that can improve our understanding of the underlying mechanisms of social functioning and the relevance of social impairments as early markers of schizophrenia, as well as potential targets for psychosocial training in 22q11.2DS are of the essence.

### Limitations

Our study showed that social impairments were more pronounced than negative and positive symptoms in our 22q11.2DS cohort where none of the participants meet the clinical diagnostic criteria within the schizophrenia spectrum. However, the cross-sectional study design is not suited to address the direction of the association between the social impairments and the subclinical symptoms (i.e., if social impairments develop before the onset of negative and positive symptoms or vice versa?). Despite this limitation, we only observed relatively low levels of current symptoms among the recruited carriers of the 22q11.2 deletion and the associations between social impairments and negative symptoms were strong and in the same direction as shown in the previous reports [42, 71]. However, the robustness of our results regarding positive symptoms may be diminished due to sample size limitations. Likewise, the uneven sex ratio and the skewed between-sex and age distributions are likely sample size dependent, and although we saw the same magnitude of social skill deficits as previously reported for 22q11.2DS [69, 70], we did not observe the usually reported superior female SRS ratings [81] in neither 22q11.2DS nor controls. Our results may also be influenced by the application of one test block only in the ERT test; nonetheless, overall, our findings of diminished emotional recognition deficits were in accordance with results reported by Jalbrzikowski et al. [42] and more recently in a group of children and adolescents with 22q11.2DS [41].

The measures of social competences, which we have applied, were not evaluated relative to a negative control measure (i.e., a cognitive function unrelated to the symptomatology) and therefore we cannot fully specify their impact on the negative and positive symptoms. The area of social functioning is complex and can be conceptualized and measured as a very important area of adaptive functioning and as autistic social and communicative deficits. Although the reliability and validity of the Danish translation of the ABAS-II has not been assessed, ample reliability and validity data are included in the ABAS-II manual [59]. Moreover, the average rating score of the 22q11.2DS cohort was more than two standard derivations below the mean of the control group indicating that everyday functioning was indeed impaired in the cases.

This study has evaluated two central aspects of social cognitive functions (ToM and emotion recognition). However, these functions may only reflect part of the multi-construct domains that they belong to. Other tasks will probably tap into overlapping or different mentalizing capacities that are equally important for performance in the social world and be implicated in negative and positive symptomatology.

Clinical case–control studies are often affected by ascertainment bias (i.e., cases being ascertained from subspecialty hospital departments and/or control individuals not being representative of the background population). However, the case group enrolled in the current study was not recruited from subspecialty hospital units. Moreover, based on the clinical profile of lifetime illnesses obtained from the Danish healthcare registries, we have previously shown that the case group is representative of the entire cohort of 22q11.2 deletion carriers known in Denmark [44]. In contrast, our control participants are probably more altruistic (i.e., willing to invest time on something that is not to the benefit of themselves) and may have stronger mental resources than the true community control would have. However, we believe that this is a general limitation of case–control studies as such.

## Conclusions

We report strong social impairments across three layers of social functioning, skills, and cognition and correlations between levels of social impairments and subclinical negative and positive symptoms. Albeit longitudinal studies are required to show if social impairments represent early disease manifestations, the associations between social impairments and subclinical positive and negative symptom severity advocate for clinical awareness of potential subclinical psychosis symptoms when parents and caregivers report substantial social impairments.

## Abbreviations

22q11.2DS: 22q11.2 deletion syndrome
ABAS_composite_: A composite score obtained from ABAS-II
ABAS-II: The Adaptive Behavior Assessment System-Second Edition
ABAS_social_: A score obtained from the social and leisure’s subdomains ABAS-II
CANTAB: Cambridge Neuropsychological Test Automated Battery
ERT: Emotion recognition task
ERT_total_: The total sum of test scores obtained in ERT
ICD: International Classification of Diseases system
IQ: Intelligence quotient
RIST: The Reynolds Intellectual Screening Test
SIPS: Structured Interview for Prodromal Syndromes
SRS: Social Responsiveness Scale
SRS_total_: The total sum of the ratings given in SRS
TASIT: The Awareness of Social Inference Test
TASIT_total_: The total sum of scores obtained in TASIT
ToM: Theory of mind
